# State Medical Library

**Published:** 1891-04

**Authors:** 


### STATE MEDICAL LIBRARY.

THE following circular letter, issued by the Legislative Committee of the Medical Society of the State of New York, explains itself:

*Dear Doctor*—At the last meeting of the New York State Medical Society, a resolution, introduced by Dr. S. B. Ward, of Albany, was unanimously carried, urging the Legislature to take steps towards establishing a State Medical Library in connection with the General Library. In keeping with this resolution a bill has been introduced in the Senate by Mr. Sheard and in the House by Dr. R. P. Bush (known as Assembly Bill No. 926), making an appropriation of five thousand dollars annually and an additional appropriation of five thousand dollars for setting apart suitable quarters, building shelves, binding, etc., for the purpose of the above resolution. The consummation of this plan will be of vast benefit to the physicians of the State, as it is intended to keep on file all the important medical exchanges, to purchase all scientific books relating to medicine, and to send catalogues to the profession generally, in order that they may obtain, for a limited time, any volume on the shelves of the Library, on application, by paying the carrying charges. In order that this bill may become a law, a united effort on the part of the profession is essential. The session is about drawing to a close, and any work in behalf of this measure must be done at once. Will you kindly, therefore, personally solicit your Senator or Member of Assembly to advocate the passage of this bill, and if a personal interview is impossible, will you write them *at once* urging favorable consideration of the measure? Very respectfully,

D. B. ST. JOHN ROOSA,
DANIEL LEWIS,
MAURICE J. LEWI,
71 Lancaster St., Albany, N. Y. *Committee on Legislation.*

At a special meeting, held March 20, 1891, the Medical Society of the County of Erie unanimously passed a resolution, introduced by Dr. Howe, asking the members of the Legislature from this county to actively urge the passage of the bill.

